# Meaning-Based Coping and Spirituality During the COVID-19 Pandemic: Mediating Effects on Subjective Well-Being

**DOI:** 10.3389/fpsyg.2021.646572

**Published:** 2021-04-15

**Authors:** Gökmen Arslan, Murat Yıldırım

**Affiliations:** ^1^Department of Psychological Counseling and Guidance, Mehmet Akif Ersoy University, Burdur, Turkey; ^2^International Network on Personal Meaning, Toronto, ON, Canada; ^3^Department of Psychology, Ağrı İbrahim Çeçen University, Agri, Turkey; ^4^Department of Psychology, University of Leicester, Leicester, United Kingdom

**Keywords:** coronavirus stress, subjective well-being, meaning-based coping, spirituality, young adults

## Abstract

The coronavirus pandemic has significantly affected the Turkish population. During the pandemic, people with high coronavirus stress are at risk of experiencing poor subjective well-being. There is no research investigating the role of meaning-based coping and spirituality in explaining the link between coronavirus stress and subjective well-being. This study examined the mediating roles of meaning-based coping and spiritual well-being in the link between coronavirus stress and subjective well-being in young adults during the COVID-19 pandemic. The sample included 427 young adults (71% female), ranging in age between 18 and 48 years (*M* = 21.06; *SD* = 2.62). Turkish young adults completed an online survey, including measures of coronavirus stress, subjective well-being, meaning-based coping, and spiritual well-being. The results indicate that greater meaning-based coping and spiritual well-being mediated decreases in the adverse impacts of coronavirus stress on subjective well-being. These results suggest that the importance of a combination of meaning-based coping and spirituality processes mitigate the adverse effects of stress on well-being during the coronavirus pandemic. Interventions focusing on meaning-based coping and spirituality in those experiencing high coronavirus stress are urgently needed to improve the mental health and well-being of young adults.

## Introduction

The coronavirus disease 2019 (COVID-19) pandemic has become a major public health problem in many countries in the world, including Turkey. It has expeditiously spread over the world, originating from Wuhan city of China in early December 2019. By March 3, 2021, the World Health Organization (2021) reported more than 113.82 million infections with 2,527,891 confirmed deaths worldwide and 2,701,588 infected cases and 28,569 deaths in Turkey. The pandemic is not only an economic, political, and social issue, but also a health issue, adversely affecting the physical, mental, and social well-being of the population (Yıldırım and Solmaz, 2020). The ongoing pandemic leads to stress, anxiety, depression, and fear among different populations (Arslan and Yıldırım, 2020; Yildirim et al., 2020b; Yıldırım and Güler, 2020a). Despite many efforts to identify the impact of the COVID-19 pandemic on well-being outcomes (Arslan et al., 2020), little is known about the mechanisms underlying the relationship between COVID-19-related risk and stress and well-being outcomes. Meaning-based coping and spirituality may help to elucidate the mechanism underlying the association between coronavirus stress and subjective well-being. The current study reports a preliminary investigation into the effects of individual factors on the subjective well-being of Turkish students with a focus on four variables, namely coronavirus risk, coronavirus stress, meaning-based coping, and spirituality.

### Coronavirus Stress and Perceived Risk

One well-known psychological aspect of the COVID-19 pandemic is coronavirus stress. The uncertain atmosphere related to COVID-19 may aggravate stress among people coping with the pandemic. For example, data from China shows that nearly 25% of the population has experienced moderate-to-severe levels of anxiety- or stress-related symptoms during COVID-19 (Qiu et al., 2020). Similarly, there is evidence of considerable stress specific to COVID-19 among the Turkish population (Yıldırım and Solmaz, 2020).

The influence of the COVID-19 pandemic on well-being and mental health outcomes has been investigated in various studies. Taken together, the results of these studies reveal a positive association between coronavirus stress and depression, anxiety, fear, burnout, trauma, and other variables related to well-being and mental health outcomes (Fisher et al., 2020; Umucu et al., 2020; Yıldırım and Solmaz, 2020; Genç and Arslan, 2021). For example, Arslan et al. (2020) establish that coronavirus stress is positively associated with depression, anxiety, and somatization. They also find the indirect mediating effects of optimism, pessimism, and psychological inflexibility on the association between coronavirus stress and psychological health. Coronavirus stress is also found to be associated with decreased meaningful living and optimism and increased depressive symptoms (Arslan and Yıldırım, 2020). Furthermore, perceived stress shows a significant positive association with coronavirus-related concern (perceived COVID-19 impact on well-being) and generalized anxiety disorders, and it demonstrates a significant negative association with satisfaction with life among Turkish students (Aslan et al., 2020). Despite this, evidence suggests that the association between stress and mental well-being outcomes is relatively complex, and the direct cause-and-effect association is unclear (Salleh, 2008). As such, examination of stress with well-being outcomes, particularly in the context of the current pandemic deserves more attention.

Perceived risk of COVID-19 is another important psychological aspect of the COVID-19 pandemic. Perceived risk plays a fundamental role in forming one’s health-related behaviors (Janz and Becker, 1984). The research on risk perception has progressively focused on beliefs, knowledge, values, and attitudes that influence not only decisions, but also behaviors of individuals with the exposure to environmental pressures (Cori et al., 2020). The risk-resilience framework suggests that risk perception in the face of difficulties elevates the occurrence of the likelihood of negative outcomes, and resilient people can turn negatives into positives (Masten, 2001). Research shows that higher risk perception is related to greater severity of disease and lower self-efficacy and mental health (Yıldırım and Güler, 2020b). Higher risk perception of coronavirus is found to be related to higher coronavirus fear, depression, anxiety, and stress (Yildirim et al., 2020a) and perceived susceptibility, worry, and disruption in daily life due to COVID-19 (Kwok et al., 2020). These results suggest that risk perception is potentially a significant psychological factor in influencing mental health in unprecedented times.

### Subjective Well-Being

The COVID-19 pandemic has exacerbated psychosocial challenges (Ahorsu et al., 2020). Subjective well-being of individuals can be negatively affected during the pandemic, which may have catastrophic consequences on people’s psychological functions in general (Yıldırım and Arslan, 2020). Subjective well-being is conceptualized as the evaluation and judgment and involves two fundamental dimensions, which are affective (positive and negative affects) and cognitive (life satisfaction; Diener, 2000; Diener et al., 2002). This study focuses on the latter dimension. People with a high level of subjective well-being experience greater satisfaction with life and positive emotions, such as happiness, contentedness, and joy. However, people with a low level of subjective well-being are dissatisfied with life and experience frequent negative emotions, such as sadness, anger, and anxiety (Diener et al., 1997, 2010). Studies show that subjective well-being is significantly associated with lowered mental health problems, such as stress, depression, anxiety, and loneliness (Yılmaz and Altınok, 2009; Beutel et al., 2010; Yildirim and Alanazi, 2018), and increased psychological resources,such as social relationships, optimism, self-efficacy, emotional regulation, adaptive coping, and self-esteem (Darling et al., 2007; Extremera et al., 2009; Özbay et al., 2012; Doğan and Eryılmaz, 2013). Previous studies also examined the associations between subjective well-being, meaning in life, and spiritual well-being. A study conducted by Aglozo et al. (2019) reports that spirituality and meaning in life positively predict positive affect and satisfaction with life, and they negatively predict negative affect. This suggests that spirituality and meaning in life contribute to and influence how people apprise their life and experience of positive and negative emotions.

### Mediating Roles of Meaning-Based Coping and Spirituality

Although the pioneering body of research has mainly examined the influence of coronavirus stress on well-being and mental health outcomes, little is known about the underlying psychological mechanism that explains how the detrimental effect of coronavirus stress on well-being can be mitigated during the COVID-19 pandemic. Researchers highlight the importance of finding effective ways to protect and improve well-being of individuals in this unprecedented period (Yıldırım and Arslan, 2020). As such, it is necessary to examine the process to mitigate COVID-19-related stressors and improve well-being. We focus on meaning-based coping and spiritual well-being, interchangeably used with spirituality, to explore the mechanism. Meaning-based coping is defined as the positive reappraisal and reinterpretation of a stressor (Wenzel et al., 2002). Meaning-based coping makes people more psychologically resilient in response to traumatic events and contributes to the protection of psychological health from the dysfunctional neural, endocrine, and immune responses to chronic stress that can result in disease (McEwen, 1998).

Research findings highlight that the absence of stress or distress does not necessarily mean that a person is optimally functioning, and instead, optimal positive psychosocial human functioning can be achieved with greater use of meaning-based coping strategies (Danhauer et al., 2005). Furthermore, evidence from qualitative and quantitative studies typically suggests that greater meaning is associated with lower stress and better coping. Meaning plays an important role in dealing with stress and trauma, which, in turn, lead to better psychological functioning and reduced distress (Halama, 2014). In addition, the mediating and moderating effects of meaning-based coping in the relationship between the number of coexisting mental health challenges and well-being are documented (Ellis et al., 2017). This suggests that cultivating meaning-based coping might be helpful in reducing distress and improving well-being in the face of adversity.

Another important mechanism that can explain the relationship between coronavirus stress and subjective well-being is that of spiritual well-being, which refers to spiritual maturity and the integral experience of an individual who is functioning as God intended (Ellison and Smith, 1991). Researchers suggest that spiritual well-being should be viewed as an ingredient of eudaimonic well-being, which, in turn, empowers the self-actualization aspect (Dierendonck and Mohan, 2006). Spiritual well-being is considered an important indicator of better well-being and mental health (Koenig, 1998; Arslan, 2021). It has been linked to a wide range of indicators of mental well-being, including greater sense of purpose, meaning in life, satisfaction with life, adaptive personality traits, and lower death anxiety (Unterrainer et al., 2014; Shirkavand et al., 2018). In a systematic review study, Unterrainer et al. (2014) report that spiritual well-being functions as a critical role in the process of recovering from mental health problems alongside acting as a protective factor against addictive or suicidal behaviors. Spiritual experiences and spiritual resources provide a sense of strength, and they are a guide to find significance or meaning in life (Wills, 2009). Therefore, it is critical to examine the association between spiritual well-being and well-being.

### Present Study

In light of the sketched literature, there are enough reasons to believe that coronavirus risk is a positive predictor of coronavirus stress, which is a negative predictor of subjective well-being, and meaning-based coping and spirituality are positive predictors of subjective well-being. Understanding the mechanisms underlying the relationships between coronavirus stress and subjective well-being would improve our understanding of these associations. In this study, we focus on meaning-based coping and spirituality and investigate the possible roles of these two variables for the associations between coronavirus stress and subjective well-being. To this end, we conducted a cross-sectional survey to examine the relationship between coronavirus risk, coronavirus stress, and satisfaction with life as a measure of subjective well-being by focusing on meaning-based coping and spirituality as possible mediators. More specifically, we tested whether (i) coronavirus risk would predict coronavirus stress; (ii) coronavirus stress would predict meaning-based coping, spirituality, and subjective well-being; (iii) meaning-based coping and spirituality would predict subjective well-being; and (iv) meaning-based coping and spirituality would mediate the relationship between coronavirus stress and subjective well-being.

## Materials and Methods

### Participants

The sample of this study comprised 427 undergraduate students from two different universities in Turkey. Participants ranged in age between 18 and 48 years (*M* = 21.06; *SD* = 2.62), and the majority of the sample was female (71%). With regard to coronavirus characteristics, the majority of the sample was not infected (95%). The data were collected during the COVID-19 pandemic throughout November 2020. A web-based survey was created using data-collection measures and demographic items. Social media was used to contact the participants. Turkish young adults completed an online survey, including measures of coronavirus stress, subjective well-being, meaning-based coping, and spiritual well-being. Before the survey items, informed consent was presented to the participants. Participation in the study was voluntary, and the survey was completed anonymously. The study was also approved by the Ağrı İbrahim Çeçen University Institutional Review Board.

### Measures

#### Coronavirus Risk

A single item was used to assess perceived coronavirus risk of young adults (“Do you feel yourself at risk due to coronavirus pandemic?”). The item was scored using a 5-point Likert-type scale, ranging from 1 (negligible) to 5 (very high).

#### Coronavirus Stress

Coronavirus stress was measured using the coronavirus stress measure (CSM), which is a 5-item self-report scale (e.g., “How often have you felt nervous and stressed because of the COVID-19 pandemic?”) developed to assess COVID-19-related stress as a stressful event (Arslan et al., 2020). All items of the measure use a 5-point Likert-type scale, ranging between 0 (never) and 4 (very often). Research indicates that the scale is psychometrically adequate and provides a strong internal reliability estimate with the Turkish sample (Arslan et al., 2020). An internal reliability estimate with the present sample was strong; see Table 1.

**Table 1:** Observed scale characteristics.

Variable	Min.	Max.	*Mean*	*SD*	Skewness	Kurtosis	α
Coronavirus risk	1	5	3.15	1.14	−0.12	−0.63	–
Coronavirus stress	0	20	12.27	5.00	−0.37	−0.55	0.90
Meaning-based coping	9	63	42.12	11.37	−0.30	−0.37	0.85
Spiritual well-being	0	20	12.41	4.96	−0.48	−0.47	0.85
Subjective well-being	5	35	18.97	7.62	0.08	−0.88	0.90

#### Subjective Well-Being

Subjective well-being was assessed using the satisfaction with life scale (SWLS), which was developed to assess people’s cognitive assessments and judgments of life (Diener et al., 1985). The SWLS is a 5-item self-report measure (e.g., “The conditions of my life are excellent”) answered based on a 7-point Likert -type scale, ranging between 1 (strongly disagree) to 7 (strongly agree). Dağlı and Baysal (2016) find that the scale has an adequate internal reliability estimate for the Turkish sample. The internal reliability estimate in this study was strong; see Table 1.

#### Spiritual Well-Being

Spiritual well-being was measured using the spiritual well-being scale (SWS) adapted from the 12-item functional assessment of chronic illness therapy-spiritual well-being (FACIT-Sp-12), which was originally developed to assess the spiritual well-being of people with cancer and chronic illnesses (Bredle et al., 2011). The SWS includes five items (e.g., “I feel a sense of purpose in my life”) with scoring based on a 5-point Likert-type scale, ranging between 0 (not at all) and 4 (very much). The psychometric properties of the SWS were investigated with the sample of this study to enhance the usability of the measure for use in research and practice in Turkish young adults. The study sample was randomly divided into two subsamples (50%). We first employed exploratory factor analysis using the principal-axis factoring extraction method (Promax rotation) with the first subsample, yielding a one-factor solution with eigenvalues > 1 (3.28) that explained 57.85% of the variance, characterized by a lack of singularity (Bartlett’s *χ*^2^ = 497.76, *df* = 10, *p* < 0.001) and adequate sample size (Kaiser-Meyer-Olkin measure of sampling adequacy = 0.86). The SWS had strong factor loadings, ranging between 0.56 and 0.85. Next, confirmatory factor analysis affirmed the unidimensional structure of the measure with the second subsample. Results from this analysis indicate a close data-model fit [*χ*^2^ = 7.15, *df* = 5, *p* = 0.21, CFI = 0.99, TLI = 0.99, RMSEA (95% CI) = 0.045 (0.00, 0.11), SRMR = 0.025], characterized by strong factor loadings (*λ* range = 0.55–0.85) and a latent construct reliability estimate (*H* = 0.89). These indicate that the SWS has psychometrically adequate properties for use in measuring the spiritual well-being of Turkish people. The internal reliability estimate with the total sample of the study was strong; see Table 1.

#### Meaning-Based Coping

The meaning-centered coping scale (MCCS; Eisenbeck et al., unpublished) was used to assess people’s meaning-based coping strategies,. It is a 9-item self-report measure (e.g., “I have faith that something positive will come out of this”), scored based on a 7-point Likert-type scale between 1 (I do not agree at all) and 7 (I completely agree). Previous research indicates that the scale is psychometrically adequate for use in many cultures, including Turkey (*α* = 0.86; Eisenbeck et al., unpublished). The internal reliability estimate in this study was strong; see Table 1.

#### Data Analyses

We performed data analyses using a two-step analytic process. As the first step of the analysis, we observed scale characteristics, and correlation analysis were conducted. The normality assumption was checked using skewness and kurtosis scores and their decision rules: skewness and kurtosis values < |1| = acceptable for normality (Field, 2009; Tabachnick and Fidell, 2013). Pearson product-moment correlation was performed to examine the association between the study variables with traditional decision rules for effect sizes: 0.10–0.29 = small, 0.30–0.49 = moderate, ≥0.50 = large (Cohen, 1988). After examining these analyses, we employed structural equation modeling to test the mediating effect of spiritual well-being meaning-based coping on the association of coronavirus stress with subjective well-being among young adults. Results from the structural equation modeling were examined using data-model fit statistics and their decision rules: Tucker-Lewis index (TLI) and comparative fit index (CFI) ≥ 0.90 = adequate and ≥ 0.95 = close model fit; standardized root mean square residual (SRMR) and root mean square error of approximation (RMSEA) ≤ 0.08 = adequate and ≤ 0.05 = close model fit (Hu and Bentler, 1999; Kline, 2015). All statistical analyses were conducted using AMOS version 24 and SPSS version 25.

## Results

### Preliminary Analyses

Observed scale characteristics indicate that all measures had relatively normal distribution with the acceptable ranges of skewness (range = −0.48 to 0.08) and kurtosis scores (range = −0.88 to −0.37) as shown in Table 1. Correlation analysis revealed that perceived coronavirus risk was positively associated with coronavirus stress, but the correlations of this variable with meaning-based coping, spiritual well-being, and subjective well-being were nonsignificant. Coronavirus stress had significant and negative correlations with meaning-based coping, spiritual well-being, and subjective well-being. Subjective well-being was also significantly and positively associated with meaning-based coping and spiritual well-being as seen in Table 2.

**Table 2:** Correlations for study variables.

Variable	1.	2.	3.	4.	5.
1. Coronavirus risk	–				
2. Coronavirus stress	0.38^**^	–			
3. Meaning-based coping	−0.06	−0.13^**^	–		
4. Spiritual well-being	−0.05	−0.24^**^	0.49^**^	–	
5. Subjective well-being	−0.06	−0.25^**^	0.42^**^	0.61^**^	–

** Correlation is significant at the 0.001 level (2-tailed).

### Structural Equation Models

Prior to testing the structural equation model, a measurement model was suggested to examine the association between latent constructs and their observed variables (Anderson and Gerbing, 1988). Coronavirus stress, coronavirus risk, subjective well-being, and spiritual well-being latent constructs were determined using their items. Overall scores of meaning-based coping were also used to define this latent structure. Findings from the measurement model indicated good data-model fit statistics (*χ*^2^ = 262.20, *df* = 111 *p* < 0.001, CFI = 0.96, TLI = 0.95, RMSEA [95% CI] = 0.057 [0.048, 0.065], SRMR = 0.035).

We next performed several structural equation models to test the role of mediators in the association between coronavirus experiences and subjective well-being of young adults. Findings from the first model, which was carried out to examine the mediating role of meaning-based coping on the effect of coronavirus experiences on subjective well-being, provided good data-model fit statistics (*χ*^2^ = 152.02, *df* = 52, *p* < 0.001, CFI = 0.96, TLI = 0.96, RMSEA [95% CI] = 0.067 [0.055, 0.080], SRMR = 0.032). The results also showed that perceived coronavirus risk was a significant predictor of coronavirus stress (*β* = 0.39, *p* < 0.001) and accounted for 15% of the variance in this variable. Coronavirus stress had a significant predictive effect on meaning-based coping (*β* = −0.15, *p* < 0.001) and subjective well-being (*β* = −0.21, *p* < 0.001). We also found a significant mediating effect of meaning-based coping (*β* = 0.40, *p* < 0.001) on the association between coronavirus stress and subjective well-being. All variables together explained 23% of the variance in subjective well-being.

We next tested the mediating effect of spiritual well-being on the relationship between coronavirus stress and subjective well-being, indicating good data-model fit statistics (*χ*^2^ = 233.84, *df* = 101, *p* < 0.001, CFI = 0.97, TLI = 0.96, RMSEA [95% CI] = 0.056 [0.046, 0.065], SRMR = 0.036). Coronavirus stress significantly predicted spiritual well-being (*β* = −0.28, *p* < 0.001) but was not a significant predictor of subjective well-being (*β* = −0.08, *p* = 0.058). Spiritual well-being significantly mediated the effect of coronavirus stress on subjective well-being (*β* = −0.28, *p* < 0.001) as seen in Table 3. Coronavirus stress explained 8% of the variance in spiritual well-being, and all variables together explained 48% of the variance in subjective well-being.

**Table 3:** Standardized indirect effects.

Mediation model	Effect	*SE*	BootLLCI	BootULCI
Meaning-based coping				
Coronavirus risk––> Meaning-based coping	−0.06	0.02	−0.11	−0.02
Coronavirus risk––> Subjective well-being	−0.11	0.03	−0.16	−0.06
Coronavirus stress––> Subjective well-being	−0.06	0.02	−0.15	−0.02
Spiritual well-being				
Coronavirus risk––> Spiritual well-being	−0.11	0.02	−0.16	−0.06
Coronavirus risk––> Subjective well-being	−0.10	0.02	−0.16	−0.06
Coronavirus stress––> Subjective well-being	−0.19	0.04	−0.27	−0.11
Meaning-based coping				
Coronavirus risk––> Spiritual well-being	−0.11	0.02	−0.16	−0.06
Coronavirus stress––> Spiritual well-being	−0.07	0.03	−0.13	−0.02
Meaning-based coping and spiritual well-being				
Coronavirus risk––> Subjective well-being	−0.11	0.02	−0.17	−0.06
Coronavirus stress––> Subjective well-being	−0.20	0.03	−0.29	−0.12

Number of bootstrap samples for percentile bootstrap confidence intervals: 10,000 with 95% bias-corrected confidence interval.

Findings from the third model, which was carried out to examine the mediating role of meaning-based coping on the effect of coronavirus experiences on spiritual well-being, yielded good data-model fit statistics (*χ*^2^ = 157.62, *df* = 52, *p* < 0.001, CFI = 0.96, TLI = 0.94, RMSEA [95% CI] = 0.069 [0.057, 0.082], SRMR = 0.039). Coronavirus stress had a significant and direct effect on spiritual well-being (*β* = −0.21, *p* < 0.001), and meaning based coping mitigated the negative effect of stress on spiritual well-being (*β* = 0.49, *p* < 0.001) as shown in Table 3. These variables together accounted for 31% of the variance in spiritual well-being of people.

**Figure 1:**
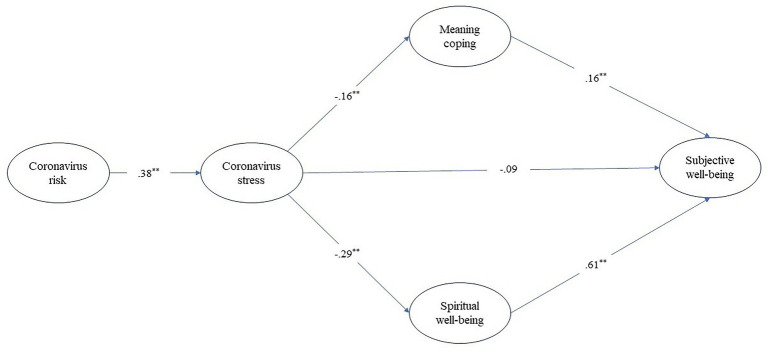
Mediation analysis of associations between the variables of the study. ^**^p < 0.001.

Spiritual well-being and meaning-based coping together were finally included in the model and examined the mediating effect of these variables together in the association between coronavirus stress and subjective well-being; see Figure 1. Findings from this analysis indicated adequate data-model fit statistics (*χ*^2^ = 369.77, *df* = 115, *p* < 0.001, CFI = 0.94, TLI = 0.93, RMSEA [95% CI] = 0.072 [0.064, 0.080], SRMR = 0.084). Coronavirus stress significantly predicted meaning-based coping (*β* = −0.29, *p* < 0.001) and spiritual well-being (*β* = −0.16, *p* < 0.001), yet the predictive effect of it on subjective well-being was nonsignificant (*β* = −0.09, *p* = 0.063). Coronavirus stress indirectly predicted subjective well-being through meaning-based coping (*β* = 0.16, *p* < 0.001) and spiritual well-being (*β* = 0.61, *p* < 0.001) as shown in Table 3. All variables together explained 45% of the variance in subjective well-being. These results indicate that the importance of the combination of meaning-based coping and spirituality processes mitigated the adverse effects of stress on well-being during the coronavirus pandemic.

## Discussion

The present study aimed to develop our understanding of the associations between coronavirus risk, coronavirus stress, and subjective well-being by focusing on meaning-based coping and spirituality as underlying mechanisms. Results support the study hypotheses. The coronavirus pandemic has become an adverse worldwide public health concern associated with various negative mental health and well-being outcomes (Arslan and Yıldırım, 2020; Yıldırım and Güler, 2020a). Therefore, coronavirus not only leads to various economic and social challenges, but also adversely influences people’s physical, mental, and social health and well-being (Yıldırım and Solmaz, 2020). Consistent with the literature (Yildirim et al., 2020a; Yıldırım and Güler, 2020b), the present evidence shows that perceived risk of coronavirus is a significant predictor of coronavirus stress in young adults. Furthermore, coronavirus stress significantly predicts spiritual well-being and meaning-based coping, which is consistent with previous research indicating that coronavirus stress is assorted with mental health problems and variables related to well-being (e.g., depression, anxiety, fear, burnout, and trauma; Fisher et al., 2020; Umucu et al., 2020; Yıldırım and Solmaz, 2020; Genç and Arslan, 2021). Perceived risk and stress may function to develop and form individuals’ stress-related behaviors (Janz and Becker, 1984) because those with high levels of perceived risk and stress are more likely to focus on feelings, thoughts, and behaviors that influence their decisions and behaviors in the face of adverse experiences (Cori et al., 2020). Theoretically, adverse circumstance-related risk perception increases the occurrence of undesirable outcomes by reducing the resilience of people, which, in turn, diminishes well-being and mental health (the risk-resilience approach; Masten, 2001). These results suggest that perceived risk and stress related to the coronavirus pandemic are potential psychological risk factors that might influence the mental health and well-being of people. Therefore, people with high levels of risk perception during the pandemic experience greater stress related to coronavirus, which, in turn, might influence their feelings, thoughts, and behaviors during the pandemic.

Most importantly, findings from the study show that meaning-based coping and spirituality explain the relationship between coronavirus stress and subjective well-being. The findings extend coronavirus research by showing that meaning-based coping and spirituality play important roles in the association between coronavirus stress and subjective well-being. The coronavirus pandemic has exacerbated psychological and social challenges (Ahorsu et al., 2020), which impact people’s subjective judgments and evaluations of their life (Diener, 2000; Kansky and Diener, 2017). Although people with high levels of subjective well-being experience more satisfaction with life and positive emotions as well as fewer negative feelings (Moore and Diener, 2019), coronavirus experiences are associated with increases in negative emotions and decreases in positive emotions (Arslan et al., 2020; Yildirim et al., 2020b). Therefore, individuals with high levels of coronavirus stress are more likely to have greater negativity and distress, which cause low subjective well-being. Additionally, social relationships are an essential source of positive mental health and subjective well-being (Kansky and Diener, 2017; Arslan, 2020; Arslan and Coşkun, 2020; Yıldırım and Çelik-Tanrıverdi, 2020). However, people have experienced various measures (e.g., quarantine) limiting their social interactions during the coronavirus pandemic to reduce the risk of infection of the virus. For example, Yildirim et al. (2020b) find a significant difference regarding psychological challenges based on COVID-19 limitations, and people who were in quarantine reported higher levels of anxiety and somatization than those who were not. People exposed to these challenges may experience greater stress, which, in turn, reduces their subjective well-being.

Despite efforts to understand the impacts of the pandemic (Arslan, 2020; Arslan et al., 2020), little is known about the mechanisms that can help to protect and promote people’s mental health and well-being in the face of the adverse impacts of this experience. Findings from the current study show that meaning-based coping and spirituality are essential sources that help to elucidate the association between coronavirus stress and subjective well-being. We first find that meaning-based coping mitigates the adverse impact of coronavirus stress on young adults’ subjective well-being. These results suggest that the positive reappraisal and reinterpretation of coronavirus experiences (Wenzel et al., 2002) makes individuals resilient in the face of the adverse impacts of the pandemic (Yildirim et al., 2020a). Using these coping strategies protects and promotes their psychological health and well-being by reducing the negative effect of stress that can result in disease (McEwen, 1998). Consistent with these results, some research emphasizes that meaning-based coping strategies help people to achieve optimal positive psychological functioning (Danhauer et al., 2005), and people with high levels of meaning report lower stress and better adaptive coping strategies in the context of challenges (Halama, 2014; Arslan and Yıldırım, 2020; Minkkinen et al., 2020). Similar to the findings of this study, Ellis et al. (2017) report the mediating effect of meaning-based coping in the association between the number of coexisting challenges and well-being among patients with advanced cancers. These results indicate that people who have high meaning-based coping might use more positive reappraisal and reinterpretation of the impacts of the pandemic and use more adaptive strategies and promotive resources that help them to build their resilience, which, in turn, improve spiritual and subjective well-being.

We also find that spiritual well-being is another important factor that mitigates the negative effect of coronavirus stress on the subjective well-being of participants. These results suggest that people with high levels of spiritual maturity (Ellison and Smith, 1991) have greater subjective well-being in spite of the predictive effect of coronavirus stress. Similar to the findings of this study, previous research reports that spiritual well-being is an important indicator of mental health and well-being, including better satisfaction with life and meaning in life (Unterrainer et al., 2014; Shirkavand et al., 2018). Spiritual resources provide a sense of strength, and they are a guide to find significance or meaning in case of adversity (Wills, 2009); thereby, spiritual well-being mitigates the impacts of adversity by playing a role in the process of recovering from psychological problems alongside acting as a protective factor against maladaptive behaviors (Unterrainer et al., 2014). High spiritual well-being might mitigate the negative effect of coronavirus stress, which, in turn, helps people to build promotive resources that foster their resilience and subjective well-being. Therefore, people with high levels of spiritual well-being are more likely to experience greater satisfaction with life and positive emotions as well as less negative affectivity during the pandemic.

The present study is not without limitations. First, the present research was a cross-sectional design. Longitudinal studies would advance our understanding of the underlying mechanisms that relate coronavirus risk and stress to subjective well-being by identifying the potential causal and temporal relationships. Second, the data were collected using self-report measures, which are considered another limitation of the study. Future studies could be conducted using various data-collection methods (e.g., qualitative) to provide additional understanding of the associations between the variables in the study. Furthermore, the convenience sampling method is also a clear limitation of this study, and therefore, future research is warranted to perform with randomly collected diverse samples, including equal genders and individuals living larger cities.

In addition to these limitations, results from this study provide important implications for research and practice that aim to improve individuals’ mental health and well-being against coronavirus experiences. The results indicate how people cope with the coronavirus pandemic that has detrimental consequences for their mental health and well-being and report that meaning-based coping mitigates the adverse impact of coronavirus stress on subjective well-being. Additionally, the findings suggest that spiritual well-being is an essential source to cultivate people’s subjective well-being by mitigating the effect of coronavirus stress. Based on these results, mental health providers could design preventions and interventions that help to improve meaning-based coping strategies, which, in turn, alleviate the effect of coronavirus stress on subjective well-being. Spiritual well-being could also be promoted through these programs, and spiritual resources might be integrated with meaning-based strategies to provide more effective coping with pandemic experiences.

In conclusion, we find that meaning-based coping and spirituality mediate the associations between coronavirus stress and subjective well-being. In addition, coronavirus risk appears to be a significant positive predictor of coronavirus stress. The present study serves to further develop our understanding of the underlying mechanisms by which coronavirus stress impacts subjective well-being. More research in this area is needed and will facilitate fully understanding the associations between coronavirus risk, coronavirus stress, meaning-based coping, spirituality, and subjective well-being.

## Data Availability Statement

The datasets generated during and/or analyzed during the current study are available from the corresponding author on reasonable request.

## Ethics Statement

The studies involving human participants were reviewed and approved by Ağrı İbrahim Çeçen University. The patients/participants provided their written informed consent to participate in this study.

## Author Contributions

GA and MY contributed to the design of the study. GA analyzed the data and wrote the materials and methods, results, and discussion sections. MY wrote the introduction. Both the authors contributed to the article and approved the submitted version.

### Conflict of Interest

The authors declare that the research was conducted in the absence of any commercial or financial relationships that could be construed as a potential conflict of interest.
